# Book Reviews

**Published:** 1799-03

**Authors:** 


### MATERIA MEDICA AND PHARMACY.

*A Praktiſche Arzneymittellehre, &c. Practical Syſtem of Materia Medica. By* JUSTUS ARNEMANN, M. D. and Profeſſor in the Univerſity of Gottingen. Third edit. enlarged and improved, 8vo. 590 pp. *Gottingen,* Vandenhoek, &c.

ALTHOUGH the author has made but few additional improvements on the ſecond edition of this work, which appeared in 1795, he has arranged the whole of its materials in a more conſpicuous manner, particularly the therapeutical properties of *mineral waters,* and the officinal *factitious airs;* each of theſe forming here diſtinct claſſes of medicinal remedies. He has further ſubjoined a new claſs of the *cooling* remedies, among which *nitre* occupies the firſt place. Beſides theſe additions, the learned Profeſſor has inriched the Materia Medica with a number of new and uſeful improvements, not included in the former edition, and of which we ſhall here only notice the *carex arenaria,* and the *calx antimonii ſulphurata,* which are very properly intitled to a place in that excellent work. The preſent volume contains what are ſtrictly called *medicinal* ſubſtances; and as the *chirurgical* remedies, according to the preface, are intended to compoſe a *ſecond* volume, ſimilar to that of the ſecond edition, this circumſtance, however, ſhould have been expreſſed ſo in the title page.

*Collections for an Eſſay towards a Materia Medica of the United States,* &c. by B. S. BARTON, *M. D.* &c. 8vo, 49 pp. *Philadelphia.* Way and Groff. 1798.

THE author of this ingenious 'Eſſay,' is unqueſtionably the firſt American, who has attempted to furniſh his countrymen with a *Materia Alimentaria*

*Alimentaria* and *Materia Medica* of their native foil. On the former, we find but few particulars, although on the latter there is an account of feveral indigenous plants of America, which promife to be ufeful in the practice of phyfic. He purfues his inquiries beyond the labours of the BARTRAMS, of COLDEN, CALM, and SCHOEPF; and appears emulous to reveal the hidden treafures of the vegetable world. Too long have the natives of the tranfatlantic continent been indebted to foreigners for genuine and fatisfactory information, relative to the plants of their own country; and, even at the prefent day, the *German* ftudent may obtain a more accurate knowledge of the trees and fhrubs of North America, from the untranflated works of WANGENHEIM, DU ROI, HEDWIG, BRIDEL, JAQUIN, &c. than they can find in any publication which has appeared in the *Englifh* language.—We cannot tranfcribe, at prefent, the different *new* vegetables which the induftrious profeffor has indicated in his Materia Medica, but we fhall endeavour to communicate them in fome future number of our Journal.

Dr. Barton has promifed, at the conclufion of this Effay, to publifh "*Some Strictures on the Claffification of Dr. Darwin,*" which we fhall take the earlieft opportunity of laying before our botanical readers.

